# VIPER regulates naive T cell activation and effector responses: Implication in TLR4 associated acute stage T cell responses

**DOI:** 10.1038/s41598-018-25549-8

**Published:** 2018-05-08

**Authors:** Subhransu Sekhar Sahoo, Belluru M. Pratheek, Vikram S. Meena, Tapas Kumar Nayak, P. Sanjai Kumar, Saumya Bandyopadhyay, Prasanta Kumar Maiti, Subhasis Chattopadhyay

**Affiliations:** 1 School of Biological Sciences, National Institute of Science Education and Research, HBNI, Bhubaneswar, Jatni, Khurda, 752050 Odisha India; 2 Abgenex India Pvt Ltd, Bhubaneswar, India

## Abstract

Naive T cells are known to express the modest level of TLR4 while it is known to go down during TCR activation. However, information towards the requirement of TLR4 signaling during TCR or mitogenic activation of naive wild-type T cells remains scanty. Here we have investigated the endogenous functional expression of TLR4 in naive mice T cells during TCR and mitogenic stimulation in presence of VIPER peptide (VP), an established inhibitor of TLR4 signaling. As expected we found that TLR4 expression goes down during TCR and mitogenic activation. Interestingly, we observed that VP treatment restores TLR4 expression on those activated T cells. Moreover, VP was found to regulate such activation of naive T cell as evident by reduction of CD25, CD69 expression, effector cytokines (IL-2, IFN-γ, TNF) production, T cell proliferation and down-regulation of T cell activation-dependent Fas (CD95), FasL (CD95L) expression. Together, our current observation highlights a possible requirement of TLR4 responses in T cells, which might have possible implication towards the pathogenic acute phase activation of naive T cells.

## Introduction

An effective immune response against an invading pathogen is coordinated by both innate and adaptive immune system. Initially, it was suggested that innate immune system works for the recognition of pathogen and adaptive immune system destroy the pathogen or pathogen-infected cells and provide long-term pathogen-specific protection. Both innate and adaptive immune system may work in an interdependent manner to efficiently protect the host from disease and infection. The first step to start an immune response is to recognize the invading pathogen. For recognition of pathogens, the innate immune system has many receptors and TLRs are the most studied one. Pathogens specific conserved structures are recognized by pattern recognition receptors (PRRs)^1^. After pathogen recognition by TLRs innate immune cells starts a cascade of signaling pathways which ultimately activates the adaptive immune system^2^.

TLR4 is one of the well studied TLR, which is expressed in the cell surface. It is expressed in the form of the homodimer, recognizes Lipopolysaccharides (LPS) from gram-negative bacteria, facilitated by CD14, Lipopolysaccharide Binding Protein (LBP) and Myeloid Differentiation Factor 2 (MD2) to activate downstream signalling^3^. TLR4 signal propagates through the cell membrane to activate Myeloid differentiation primary response gene (88) (MYD88) dependent pathway or TIR-domain-containing adapter-inducing interferon-β (TRIF) dependent pathway in cytoplasm further cascades into nucleus resulting in activation of genes of pro-inflammatory cytokines^4^.

Classically TLRs are known to be most efficient modulators of innate immunity. However recent evidence proposes an important role of TLRs in modulating adaptive immune response. There were certain suggestions that TLR4 is polarised towards TH1 response of antigen presenting cells^2,5^. Several studies suggest the expression and functional significance of TLRs in T cells^6,7^. Naive mouse T cells are found to express detectable level of TLR4 expression, while it may go down during TCR activation without having a direct responsiveness of LPS on T cells^8,9^. However, LPS has been shown to modulate the efficacy of regulatory T cells^10^. Moreover, it has been reported that T cell adhesion and chemotaxis could be regulated by LPS^11^. It has been proposed that TLR2 and TLR4 signaling could upregulate suppressor of cytokine signaling 3 (SOCS3) expression and downregulate T cell effector function^12^. Moreover, an apparent contrasting role of differential TLR4 signaling has been reported towards regulating inflammation associated with Tregs and CD4^+^ T cell responses^9,13^. Recently, CD8^+^ T cells from a specific cohort of rheumatoid arthritis (RA) patients, unlike naive healthy donors and Systemic Lupus Erythematosus (SLE) patients, have been shown to express elevated surface TLR4 expression and also found to respond upon LPS treatment^14^. However, the requirement of TLR4 responses towards TCR or mitogen directed acute stage T cell activation and effector function in wild-type naïve T cell population, if any, is not well reported.

Viral inhibitory peptide for TLR4 (VIPER) is an inhibitory peptide (11 aa long) specific for TLR4 derived from the A46 protein of vaccinia virus. It interacts with adaptor proteins: MyD88 adaptor-like (Mal) and TRIF-related adaptor molecule (TRAM) to inhibit TLR4-mediated MAPK and transcription factor activation. It has been shown that VIPER is able to inhibit TLR4 mediated immune response in innate immune cells such as macrophages^15^. In another study, VIPER inhibited inflammatory responses elicited by *Mycoplasma pneumoniae* in mouse macrophage suggesting a role of TLR4 in the *M. pneumoniae* mediated inflammatory responses^16^. Furthermore, treatment of mouse neuronal cells with VIPER was found to completely block TLR4 mediated chemokine (C-X-C motif) ligand 1 (CXCL1) expression and its release. It also inhibited intercellular adhesion molecule 1 (ICAM-1) and vascular cell adhesion molecule (VCAM-1) expression on endothelial cells, and induced infiltration of neutrophils across the endothelial monolayer^17^. Treatment of VIPER through intracerebroventricular route in hypertensive rat leads to reduced circulating norepinephrine levels which resulted in inhibition of delayed progression of hypertension and improvement of cardiac hypertrophy and function. Further, it reduced myocardial TNF-α, IL-1β, iNOS levels, NF-κB activity, and altered renin-angiotensin system components significantly^18^. This reveals the role of TLR4 in brain attenuation of angiotensin II-induced hypertensive response. Additionally, *in vivo* treatment of VIPER is also found to protect the rat from acute kidney injury mediated by LPS driven TLR4 stimulation^19^. It also facilitates improved glomerular filtration rate, elevated renal blood flow, and a reduced renal vascular resistance, reduction in the rate of production of free radicals (reactive oxygen species and superoxide), proinflammatory cytokine, and acute kidney injury markers^20^. Recently it has been shown that VIPER regulates LPS mediated CD8^+^ T cell inflammatory cytokine responses from a cohort of RA patients unlike the T cells from healthy controls and SLE patients^14^. Collectively, these studies show the role of VIPER in understanding various TLR4 driven response in different cells and systems. However, the role of VIPER towards naive T cell activation and its regulation associated with effector function is yet to be explored.

In this study, we have used VIPER, as an established TLR4 signaling inhibitor and found that it may regulate the activation and effector function during TCR and mitogenic stimulation of naive T cells derived from mice. To our knowledge, this is the first report which shows that VIPER driven inhibition of TLR4 signaling may regulate activation and effector function of naive T cells from wild-type mice, which might have implication towards altered immune responses during acute stage T cell activation processes.

## Results

### VIPER suppresses TNF production in Raw 264.7 cells and mouse splenocytes

**Figure 1:**
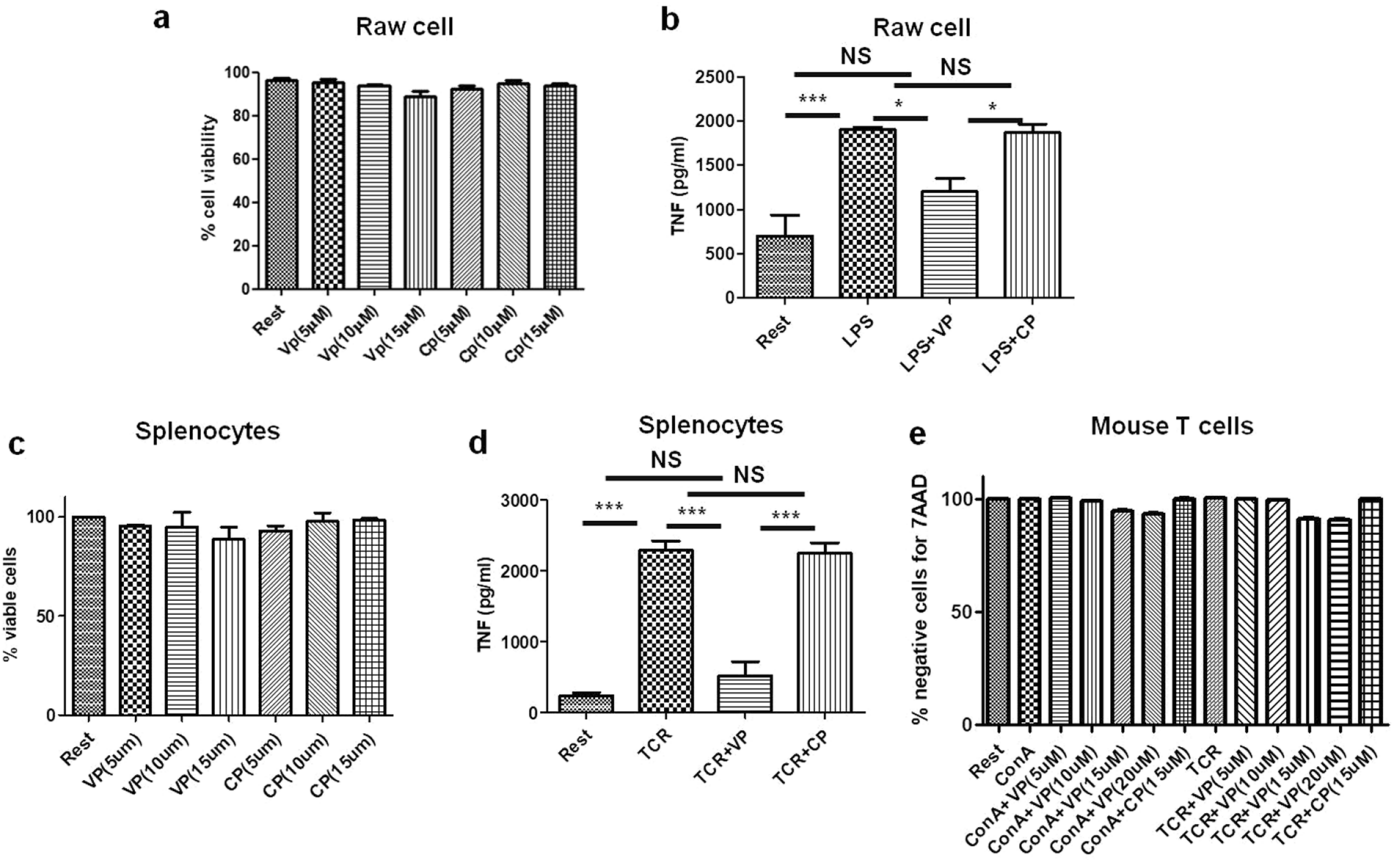
Cell viability and suppression of TNF secretion in Macrophages and splenocytes. (**a**) Dose kinetic study show Raw 264.7 mouse macrophage cells are viable at high concentration of VIPER i.e.15 µM. Cell viability was done by trypan blue exclusion method. (**b**) VIPER suppresses TNF secretion in Raw cells during LPS mediated activation. (**c**) Mouse splenocytes were treated with different doses of VIPER and cell viability is studied using trypan blue exclusion method. At 5 and 10 µM concentration, cells are viable. (**d**) VIPER suppresses TNF in splenocytes activated with TCR. (**e**) Percent negative cells for 7AAD shows the viability of purified mouse T cells with VIPER and Control Peptide in the presence of TCR/ConA stimulation. Data shown are representative of three independent experiments and Bars represent the mean ± SEM. VP: VIPER, CP: Control Peptide. *P < 0.05, **P < 0.01, ***P < 0.001.

First, we did trypan blue exclusion assay with different dose of VP and Control Peptide (CP) in Raw 264.7 cells to find out the concentration at which cells are viable. We found that cells are viable at concentrations up to 15 µM of VP and CP (Fig. 1a). VP is an established TLR4 specific inhibitor reported to suppress LPS mediated TNF secretion in macrophages^15,16^. To ensure such effect of VP in our set up on macrophage (Raw 264.7 mouse macrophage cell line) we pre-incubated Raw cells with 5 µM^15^ of VP and CP for 1 h and then treated them with 250 ng/ml LPS. The cell-free supernatant was collected after 8 h and then ELISA was done for TNF. VP treated Raw cells show a significant reduction in TNF secretion with respect to cells treated with only LPS in combination with CP as reported previously^15^ (Fig. 1b). Splenocytes produce TNF upon TCR mediated stimulation. So we want to know the effect of VP on splenocytes. For that first we did cell viability assay by using Trypan Blue exclusion method with different dose of VP and found at 5 to 15 μM concentration cells are viable (Fig. 1c). Cell viability was also studied using 7AAD for purified mouse T cells with VP and CP in presence of TCR/ConA stimulation (Fig. 1e). We found that up to 10 μM of VIPER concentration nearly 100% cells are negative for 7AAD and up to 15 μM VIPER concentration more than 90% of cells are negative for 7AAD (Fig. 1e). As most of the cells are viable at 10 μM concentration of VP we used 10 μM of VP and CP for our further experiments. To study the effect of VP on splenocytes for TNF production BALB/c mouse splenocytes were pre-incubated with 10 μM VP and CP for 2 h. Then cells were stimulated with TCR. After 36 h cell culture supernatant was collected and ELISA was done for TNF secretion. VP treated splenocytes shows a significant reduction in TNF production when compared with cells treated with TCR or TCR along with CP (Fig. 1d). These data suggest that VP can suppress TNF production by naïve mouse splenocytes during TCR activation. 10 µM of VP and CP are used as an optimum concentration for suppression of T cell activation for all further experiments.

### Differential surface expression of TLR4 in ConA, TCR, and VP treated cells

**Figure 2:**
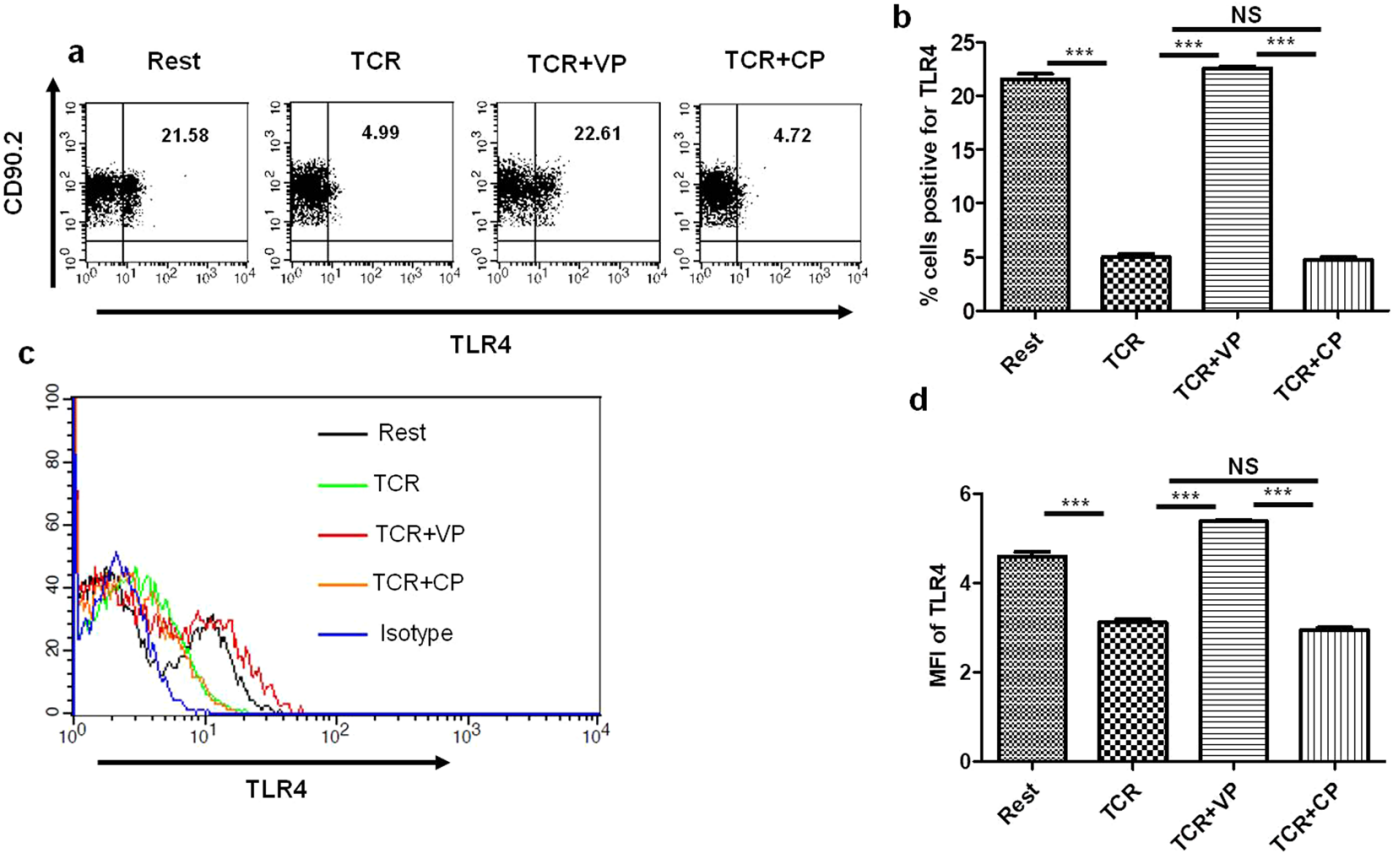
Differential surface expression of TLR4 during T cell activation. (**a**) Flow cytometry dot plot showing frequency of T cells (% positive cells) expressing TLR4 is significantly reduced during TCR mediated T cell activation and VIPER treated cells showed restoration of TLR4 expression. (**b**) Bar graphs showing % positive cells expressing TLR4 from three independent experiments. (**c**) MFI plot showing TLR4 MFI is reduced in TCR activated cells whereas VIPER treated cells show restoration of TLR4 expression. (**d**) Bar graph representing MFI data of TLR4 expression from three independent experiments. Data shown are representative of three independent experiments and Bars represent the mean ± SEM. *p < 0.05, **p < 0.01, ***p < 0.001.

Previous studies have suggested the expression of TLR4 on the mouse as well as human T cells^6–9^. To investigate the differential expression of TLR4, if any, we surface stained the purified mouse splenic T cells treated with VP, CP along with TCR for 36 h with TLR4 antibody and analyzed with flow cytometry. We found that 21.5 ± 0.5% of resting T cells express TLR4 on cell surface whereas during TCR mediated stimulation TLR4 expression (only 4.99% ± 0.28 cells) on the cell surface is going down significantly (Fig. 2a). Interestingly TLR4 expression in VIPER treated cells remained similar as compared to resting cells (22.61 ± 0.18%) whereas CP treated cells showed a reduction (4.72 ± 0.27%) in TLR4 surface expression at par with TCR treated cells (Fig. 2a,b). Similar results were observed with MFI data of TLR4 expression in VIPER treated cells (Fig. 2c,d). These data suggest that VP may restore TLR4 expression during activation of naïve T cells.

### VIPER down-regulates activation of naive T cells

**Figure 3:**
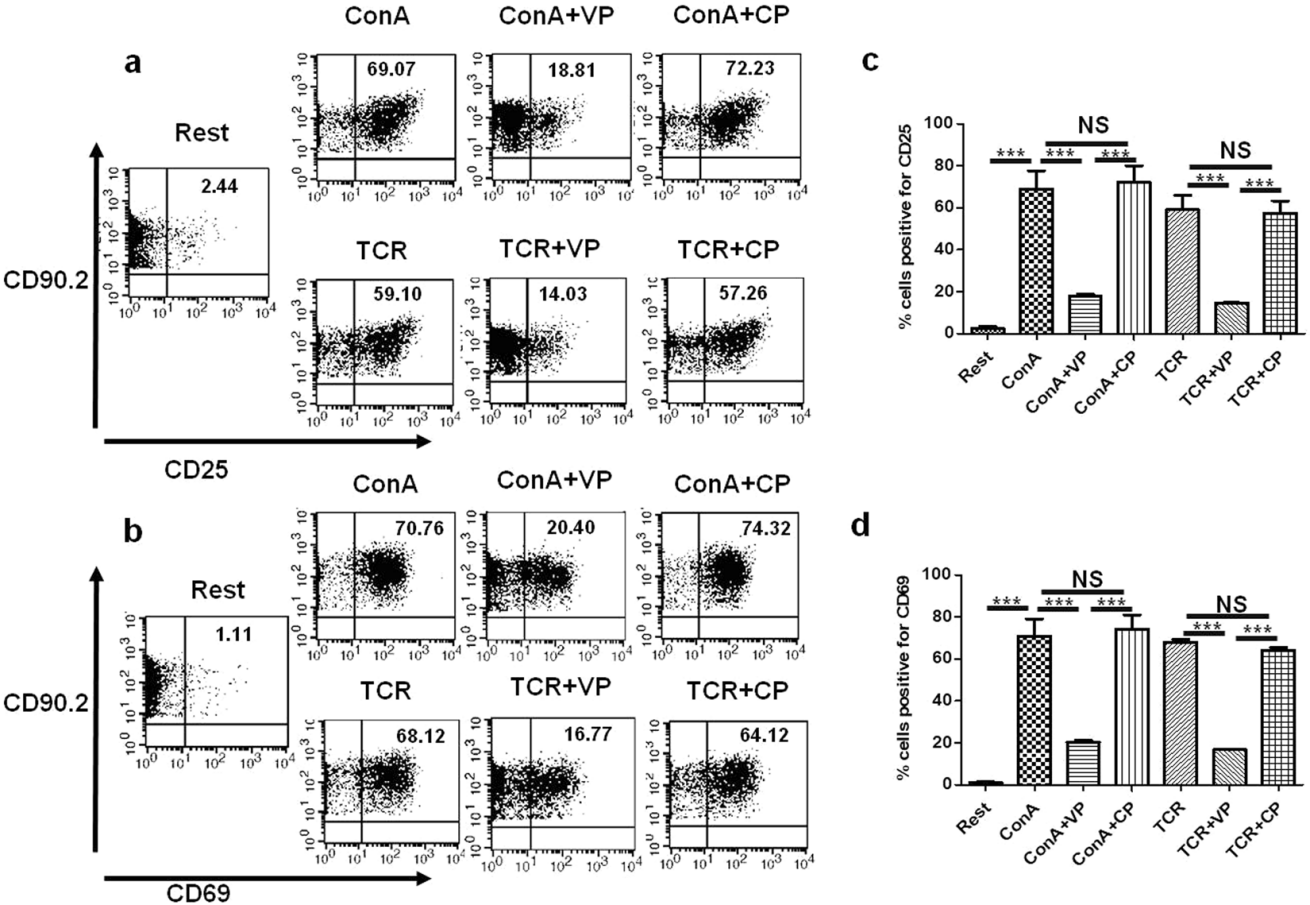
VIPER suppress the expression of T cell activation markers during T cell activation. (**a**) Flow cytometry dot plot showing CD25 surface expression (% positive cells) is significantly reduced in VIPER treated cells during ConA or TCR mediated T cell activation. (**b**) Similarly, the CD69 expression is also decreased in VIPER treated cells in comparison with cells activated only with ConA or TCR. (**c**,**d**) Bar graphs showing % positive cells expressing CD25 and CD69 respectively from three independent similar experiments Data shown are representative of three independent experiments and Bars represent the mean ± SEM. *p < 0.05, **p < 0.01, ***p < 0.001.

After observing that VIPER down regulates TNF production in splenocytes and regulates surface expression of TLR4 in purified T cells during TCR stimulation we wanted to know whether it has a direct effect on T cells activation or not. Purified T cells were pre-incubated with VP and CP for 2 h and then treated with ConA and TCR for 36 h. Cells were harvested after 36 h and stained for surface expression of T cell activation markers CD25 and CD69. Stained cells were acquired and CD90.2 gated cells were analyzed. After ConA activation 69.07 ± 8.58% cells were positive for CD25 (Fig. 3a) and 70.76 ± 8.34% cells were expressing CD69 (Fig. 3b). However, in VP treated T cells only 18.81 ± 0.83% and 20.40 ± 0.98% cells were positive for CD25 (Fig. 3a) and CD69 (Fig. 3b) respectively. Similarly in TCR treated T cells 59.10 ± 6.94% and 68.12 ± 1.28% cells are positive for CD25 (Fig. 3a) and CD69 (Fig. 3b) respectively, whereas VP treated cells showed significant reduction in expression of activation marker CD25 (14.03 ± 0.05% cells, Fig. 3a) and CD69 (16.77 ± 0.08% cells, Fig. 3b). CP treated cells showed no significant difference in CD25 (Fig. 3a) and CD69 (Fig. 3b) expression as compared to ConA and TCR treated cells. This observation suggests that VP may regulate naive T cell activation.

### VIPER down-regulates T cell effector cytokine production during TCR and ConA stimulation

**Figure 4:**
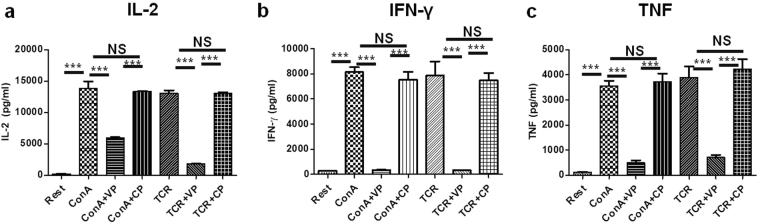
VIPER suppress T cell effector cytokine response. Cytokine ELISA data in pg/ml for IL-2 (**a**), IFN-γ (**b**) and TNF (**c**) shows VIPER treated T cells produce less inflammatory cytokines as compared to cells treated with ConA, TCR or with Control Peptide. Data shown are representative of three independent experiments and Bars represent the mean ± SEM. *p < 0.05, **p < 0.01, ***p < 0.001.

Naive T cells are known to produce effector cytokines during TCR and ConA stimulation^21^. Here we studied the production of T cell effector cytokines IL-2, IFN-γ and TNF upon VP and CP treatment during TCR and ConA mediated activation. Cytokine ELISA was done with cell culture supernatants collected from VP and CP pretreated cells stimulated with ConA or TCR for 36 h. We have observed that signature T cell effector cytokines IL-2 Fig. 4(a), IFN-γ Fig. 4(b), and TNF Fig. 4(c) secretion were significantly downregulated in VP treated cells as compared to CP treated cells.

### TLR4 inhibitory peptide VIPER inhibits T cell proliferation

**Figure 5:**
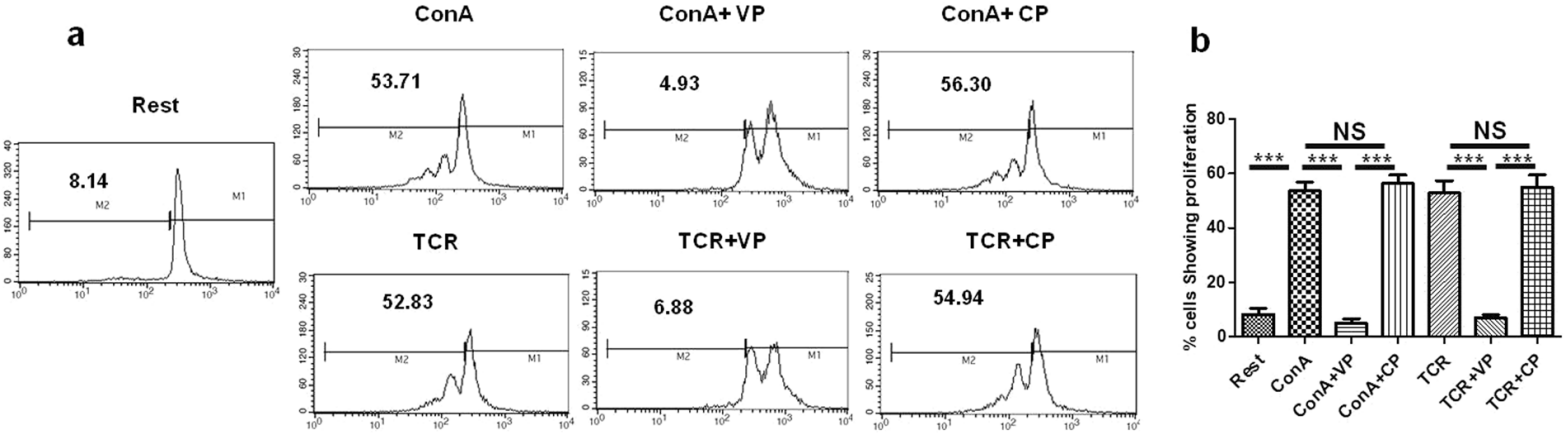
VIPER suppress T cell proliferation. (**a**) Flowcytometry histograms showing proliferation status of purified T cells from BALB/c mice which are untreated or stimulated with ConA, ConA + VIPER, ConA + Control Peptide, TCR, TCR + VIPER and TCR + CP respectively. (**b**) Bar diagram showing % cells proliferating from three independent similar experiments. Data shown are representative of three independent experiments and Bars represent the mean ± SEM. *p < 0.05, **p < 0.01, ***p < 0.001.

TCR mediated stimulation is supposed to be followed by IL-2 secretion which in turn helps in T cell proliferation^22^. As VP down-regulated IL-2 secretion we are curious to know if it has any effect on T cell proliferation. We first labeled the naive T cells with CFSE and then treat them with different conditions as shown in Fig. 5(a) for 96 h. We observed that T cells treated with VP were showing significantly less proliferation as compared to cells treated only with ConA or TCR (Fig. 5b). 53.71 ± 3.17% T cells showed proliferation after ConA treatment whereas only 4.93 ± 1.67% cells showed proliferation when pretreated with VP before ConA treatment (Fig. 5a). Similarly, 52.83 ± 4.58% TCR treated cells showed proliferation whereas 6.88 ± 1.28% cells pretreated with VP were proliferating upon TCR stimulation (Fig. 5a). CP treated cells showed no significant difference in proliferation as compared to cells stimulated with only ConA or TCR.

### VIPER regulates T cell activation directed Fas and FasL expression

**Figure 6:**
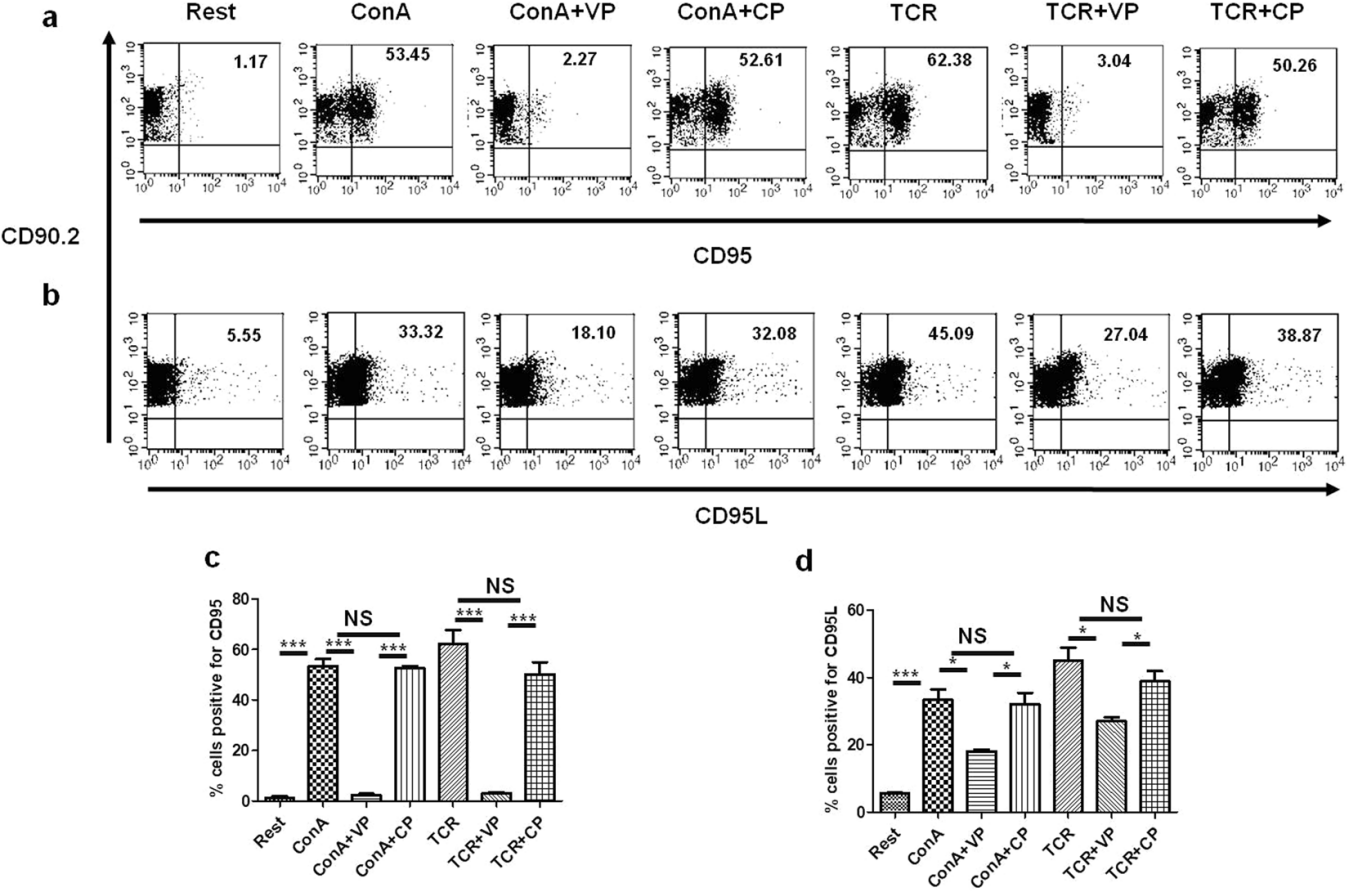
VIPER regulates induction of Fas (CD95) and FasL (CD95L) during T cell activation. Flow cytometric dot plot showing expression of CD95 (**a**) and CD95L (**b**) in T cells treated with VIPER and CP followed by ConA and TCR mediated activation. VIPER treated cells showed significantly reduced expression (% positive cells) of CD95 and CD95L as compared to cells treated with only ConA /TCR. Bar diagram showing % cells expressing (**c**) CD95 and (**d**) CD95L from three independent similar experiments. Data shown are representative of three independent experiments and Bars represent the mean ± SEM. *p < 0.05, **p < 0.01, ***p < 0.001.

Optimum T cell activation is followed by Activation-Induced Cell Death (AICD) to maintain T cell homeostasis^23^. As we found that VP suppresses T cell activation we were interested to study whether it has any effect on the induction of AICD or not. So we studied the expression of CD95 and CD95L in VP treated naive T cells during TCR and ConA mediated activation. We found that after ConA or TCR mediated activation CD95 and CD95L expressions were induced. However, the cells treated with VP showed significant down-regulation in both CD95 and CD95L expression after ConA or TCR mediated activation (Fig. 6). 1.17 ± 0.52% resting cells express CD95 whereas during ConA and TCR mediated activation it goes up to 53.45 ± 2.71% and 62.38 ± 5.25% respectively (Fig. 6a). Interestingly T cells treated with VP shows significantly reduced expression of CD95 (Fig. 6c). Only 2.27 ± 0.57% and 3.04 ± 0.48% VIPER treated T cells were expressing CD95 with ConA and TCR respectively (Fig. 6a). Similarly, the CD95L expression is upregulated during T cell activation. In resting condition 5.55 ± 0.26% cells were expressing CD95L whereas during activation with ConA and TCR it increased up to 33.32 ± 3.1% and 45.09 ± 3.7% (Fig. 6b). VP treated cells showed significantly reduced expression of CD95L (Fig. 6d). In VP pretreated cells 18.1 ± 0.54% cells expressed CD95L with ConA and 27.04 ± 1.16% with TCR (Fig. 6b). Decreased surface expression of CD95 and CD95L in VP treated T cells suggest that it may regulate the induction of AICD.

## Discussion

Here we have investigated the role of VIPER, a TLR4 specific signaling peptide inhibitor towards exploring the possible requirement of TLR4 in naive T cell activation *in vitro*. TLR4 has been reported mostly on antigen presenting cells and their possible modulation can alter the outcome of the cell-mediated immunity^2,5^. Functional expression of TLR4 on various subsets of T cells in different disease condition and experimental models has been reported^9,14,24,25^. TLRs have been shown to regulate innate and adaptive immune systems in different disease conditions like autoimmune diseases and infectious diseases. It’s possible that in autoimmune diseases prolonged chronic inflammation persists as compared to acute inflammatory responses^26,27^. The role of TLR4 and TLR2 has been reported and reviewed in different autoimmune conditions like RA and SLE^28–31^. TLR4 had been shown to aggravate RA in mouse model whereas TLR2 has been reported to be associated with protective role^28^. However, those reports are illustrated with different experimental models and suggest a differential functional role of TLR4 on T cells. Moreover, some reports also suggest there is no such direct role of LPS, a TLR4 specific ligand, on naive T cells^10^. Direct modulation of TLR4 signaling in wild-type naive T cells of mouse origin or from T cells from healthy human donors is necessary to predict the requirement of TLR4 signaling towards T cell activation if any.

TCR mediated activation can down-regulate TLR4 expression in T cells as compared to untreated cells^8,9^. We have also found similar down-regulation of TLR4 surface expression on T cells during TCR driven activation. Moreover, we observed that VP and TCR treated T cells showed a similar level of surface expression of TLR4 as untreated cells indicating that VP was actively resisting the TCR induced down-regulation of TLR4 in T cells. In presence of a scrambled peptide (CP), down-regulation of TCR-driven TLR4 expression remained unaffected. Several reports suggest some important roles of MyD88, a TLR4 associated signaling adapter protein, towards T cell effector function^32–35^. However, a direct role of TLR4 responsiveness towards activation of wild-type naive T cells remains scanty. Accordingly, we further studied the effect of VP towards effector function during TCR and mitogenic activation on naive T cell. We observed that VP treated naive T cells showed significantly reduced expression of T cell activation marker CD25 and CD69 as compared to untreated cells upon TCR or mitogenic stimulation. Similarly, we observed that upon TCR or mitogenic stimulation, effector cytokine secretion is decreased in VP treated cells. Together these results suggest TLR4 signaling might have some regulatory function during naive T cell activation. Additionally, our results showed TCR or mitogen mediated naive T cell proliferation can be regulated by VP. VP treated T cells showed reduced proliferation as compared to cells treated only with TCR or ConA. Optimum T cell activation is followed by clonal propagation and then activation-induced cell death (AICD) to maintain homeostasis of T cell population. AICD is mediated by expression and involvement of CD95 (Fas) and CD95L (Fas ligand) on T cells^23,36^. Interestingly we observed that VP regulates induction of CD95 and CD95L expression during T cell activation. This proposes that VP down-regulates T cell activation which in turn may regulate the expression of CD95 and CD95L.

In brief, our current investigation suggests that there might be a requirement of TLR4 signaling during naive T cell activation and effector function. Here we have shown VIPER driven regulation of TLR4 responses towards regulating activation, proliferation, effector cytokine production, Fas and FasL induction of naive T cells, which might have possible implication towards the pathogenic acute phase activation of T cell responses.

## Materials and Method

### Mice

Female or male BALB/c mice of 8–10 weeks old were used in the experiments. Mice were from animal facility of National Institute of Science Education and Research (NISER). All experiments using animals were approved by NISER’s Institutional Animal Ethics Committee according to the guidelines set by Committee for the Purpose of Control and Supervision of Experiments on Animals (CPCSEA).

### Chemicals, Antibodies and other Reagents

Concanavalin A (ConA) was bought from Sigma Aldrich and LPS was from HiMedia. TLR4 Peptide Inhibitor Set: VIPER and Control Peptide were obtained from Novus Bio, CO, USA, used as TLR4 signaling inhibitor. The following antibodies were used: anti-mouse CD90.2 APC from Neuprocells, CD25, CD69, CD95 and CD178 (CD95 ligand) are from BD Biosciences (San Diego, CA); TLR4-Alexafluor 488 was from eBioscience, αCD3 antibody used for the functional assay was from BD Bioscience and αCD28 was from Neuprocells. T cell enrichment kit and CFSE for T cell proliferation assay were from Invitrogen. ELISA kits from BD Biosciences were used for sandwich ELISA of TNF, IL-2, and IFN-γ.

### Preparation of mouse splenic T cells

Splenocytes from BALB/c mouse spleen was isolated as reported earlier^21,37^. The spleen was disrupted through a 70μm cell strainer. RBCs were lysed by RBC lysis buffer and cells were washed with 1XPBS and suspended in complete RPMI. T cell purification was done by using untouched mouse T cell isolation kit from Invitrogen according to instructions given by the manufacturer. In brief, splenocytes were resuspended in isolation buffer and incubated with biotinylated antibody for 20 mins. Then cells were washed in excess isolation buffer and incubated with streptavidin-conjugated magnetic beads for 15 min and then placed on a magnet for 2 mins. Enrichment of the T cells was evaluated by flow cytometry and found to be ≥96%.

### Cell culture

Purified T cells prepared from spleens of mice were pretreated with VP (10 µM/ml) or CP (10 µM/ml) in complete RPMI and incubated at 37 °C, 5% CO_2_ humidified incubator for 2 h followed by stimulation with ConA (5 µg/ml) or TCR. 1.5 × 10^6^cells/ml cells were cultured in 48-well tissue culture plates (500 μl/well) in RPMI media supplemented with 10% FBS at 5% CO_2,_ 37 °C for 36 h in humidified incubator followed by flow cytometric analysis and the cell culture supernatants were kept in −80 °C for ELISA for the respective samples.

### Cell viability assay

Splenocytes were incubated with different doses of VP and CP for 36 h. Cell viability was studied by trypan blue exclusion method. Purified mouse T cells were incubated with different doses of VP and CP along with TCR/ConA stimulation for 36 h. Cells were harvested and washed after 36 h followed by incubation with 7AAD for 15 mins. Cells were then acquired and analyzed in BD FACS Calibur using Cell Quest Pro software. 7AAD negative cells were considered as live cells.

### Flow Cytometry

Flow cytometric study of T cells was carried out as reported previously^21,37^. In brief, Cells were incubated with fluorochrome-conjugated antibodies for 30 mins and then washed two times and resuspended in staining buffer. Cells were acquired using BD FACS Calibur and live gated population was analyzed by Cell Quest Pro software.

### ELISA

T cells isolated from BALB/c mice were pretreated with the desired concentration of VP and CP for 2 h and then stimulated ConA or TCR (anti-CD3/CD28mAbs: 1 μg anti-CD3 mAb as plate bound and 2 μg/ml soluble form of anti-CD28 mAb)^35^ and incubated for 36 h. Cell-free supernatants were collected at 36 h after culture and stored in −80 °C until further use. ELISA was done as described before^21,38^ for IL-2, IFN-γ, and TNF according to manufacturer’s instructions and reagents provided (BD OptEIA ELISA kit Sets). Samples were plated in duplicate.

### T cell Proliferation Assay

Purified T cells are incubated with 5 µM CFSE from Invitrogen (CellTrace^TM^ CFSE Cell Proliferation Kit, Cat no. C34554) in 1X PBS for 10 minutes at room temperature and then washed three times with RPMI media supplemented with 10% FBS. Then cells were pretreated with the desired concentration of VP and CP for 2 h. After 2 h cells were stimulated with ConA or TCR and incubated for 96 h. Cells were harvested and washed with FACS buffer. Treated cells were acquired with BD FACS Calibur and analyzed in Cell Quest Pro software.

### Statistics

Data are presented as means ± standard errors of the means (SEM) of three similar independent experiments. Differences between two groups were determined by One way ANOVA using GraphPad Prism 5.03 software. A *p*-value of <0.05 was considered significant between groups.

### Data availability

All data generated during this study are included in this published article.
